# Barriers to and facilitators for screening women for intimate partner violence in surgical fracture clinics: a qualitative descriptive approach

**DOI:** 10.1186/1471-2474-14-122

**Published:** 2013-04-05

**Authors:** Sheila Sprague, Marilyn Swinton, Kim Madden, Rukia Swaleh, J Carel Goslings, Brad Petrisor, Mohit Bhandari

**Affiliations:** 1 Department of Clinical Epidemiology & Biostatistics, McMaster University, 293 Wellington St. N Suite 110, Hamilton, ON L8L 8E7, Canada; 2 Trauma Unit, Department of Surgery, Academic Medical Center, University of Amsterdam, Meibergdreef 9, Amsterdam, AZ 1105, The Netherlands; 3 Division of Orthopaedic Surgery, Department of Surgery, McMaster University, 293 Wellington St. N Suite 110, Hamilton, ON L8L 8E7, Canada

**Keywords:** Intimate partner violence (IPV), Musculoskeletal injuries, Barriers, Screening

## Abstract

**Background:**

Intimate Partner Violence (IPV) is a major health issue that involves any physical, sexual or psychological harm inflicted by a current or former partner. Musculoskeletal injuries represent the second most prevalent clinical manifestation of IPV. Health care professionals, however, rarely screen women for IPV. Using qualitative methods, this study aimed to explore the perceived barriers to IPV screening and potential facilitators for overcoming these barriers among orthopaedic surgeons and surgical trainees.

**Methods:**

We conducted three focus groups with orthopaedic surgeons, senior surgical trainees, and junior surgical trainees. A semi-structured focus group guide was used to structure the discussions. Transcripts and field notes from the focus groups were analyzed using the qualitative software program N’Vivo (version 10.0; QSR International, Melbourne, Australia). To further inform our focus group findings and discuss policy changes, we conducted interviews with two opinion leaders in the field of orthopaedics. Similar to the focus groups, the interviews were digitally recorded and transcribed, and then analyzed.

**Results:**

In the analysis, four categories of barriers were identified: surgeon perception barriers; perceived patient barriers; fracture clinic barriers and orthopaedic health care professional barriers. Some of the facilitators identified included availability of a crisis team; development of a screening form; presence of IPV posters or buttons in the fracture clinic; and the need for established policy or government support for IPV screening. The interviewees identified the need for: the introduction of evidence-based policy aiming to increase awareness about IPV among health care professionals working within the fracture clinic setting, fostering local and national champions for IPV screening, and the need to generate change on a local level.

**Conclusions:**

There are a number of perceived barriers to screening women in the fracture clinic for IPV, many of which can be addressed through increased education and training, and additional resources in the fracture clinic. Orthopaedic health care professionals are supportive of implementing an IPV screening program in the orthopaedic fracture clinic.

## Background

Intimate Partner Violence (IPV) is described by the American Medical Association as “a pattern of coercive behaviors that may include repeated battering and injury, psychological abuse, sexual assault, progressive social isolation, deprivation and intimidation” [1]. Injuries associated with IPV often require treatment provided by orthopaedic surgeons [2]. Canadian orthopaedic surgeons may not recognize the extent that IPV affects the patients seen in their clinics; an overwhelming 87 percent who participated in a national study believed that female victims of IPV accounted for less than one percent of patients in their care [3]. A recent prevalence study found that one third of women attending two fracture clinics for an orthopaedic injury had experienced physical, emotional, and/or sexual abuse within the last 12 months [4]. This rate of IPV is much greater than the orthopaedic surgeons estimated [3], and provides a rationale for IPV screening and support programs in orthopaedic clinics.

To address the low rates of screening, previous studies have explored barriers to IPV screening among various health care professionals such as emergency department health care workers, obstetricians/gynecologists, family physicians, internists and health care staff in family planning organizations [5-8]. A recent systematic review of 22 studies investigating barriers to IPV screening reported by health care professionals described five categories of barriers: 1) patient-related barriers; 2) health care provider fears; 3) lack of resources; 4) personal barriers; and 5) health care provider misconceptions [8]. Across the included studies, the most commonly cited barriers to screening for IPV were personal discomfort with the issue of IPV, lack of time, and lack of knowledge about IPV [8]. This review did not find any studies that comprehensively examined the perceived barriers to IPV screening among health care professionals who treat patients in the orthopaedic fracture clinic setting [8].

This research aimed to address this gap in the literature by exploring perceived barriers to IPV screening in the orthopaedic fracture clinic and by identifying potential facilitators for addressing these barriers among orthopaedic surgeons and surgical trainees (senior and junior orthopaedic residents).

## Methods

### Qualitative method and rationale

This research was conducted using the qualitative descriptive approach, a qualitative research method which aims to provide a descriptive summary of the research organized in a way that best reflects the data. This method is described by Sandelowski [9] as being valuable when straight descriptions are required to provide answers to questions of special relevance to practitioners and policy makers.

The authors are qualified to conduct this study because we have an interdisciplinary mix of expertise in qualitative methods, intimate partner violence, orthopaedic surgery, and trauma surgery. Some authors had preconceived ideas of what some of the barriers may be and others did not. The mixture of areas of expertise contributed to the multiple perspectives needed to effectively analyze the information of interest in an appropriate and holistic context.

### Data collection

Data were collected through focus groups comprised of orthopaedic surgeons and orthopaedic surgical trainees. Focus groups are generally recommended for qualitative descriptive studies as they typically provide a broad range of information about experiences [9]. The opportunity for interactive discussion during the focus groups enhanced the ability to collect in-depth data on the perceived barriers to and facilitators for screening for IPV in the orthopaedic fracture clinic. We conducted three separate focus groups – one with orthopaedic surgeons, one with senior surgical trainees, and one with junior surgical trainees. When transcribing data, we did not record the names of any participants to preserve their privacy and confidentiality. We assigned each participant a code, such as “Surgeon 1” or “Junior resident 2”. The codes were kept in a secure location and only one member of the team had access to the codes.

### Sampling

Sampling for this study was purposeful with an emphasis on maximum variation sampling. The goal of purposeful sampling is to select participants who provide “information-rich” cases, that is, participants who provide data that will allow us to learn in-depth about the phenomenon of interest [10]. In this study, we purposefully sampled to include variation in health care professional type (i.e. surgeons and surgical trainees) because we believe that this variable has the potential to influence experiences with and perceptions about screening for IPV. The research coordinator (KM) emailed invitations to the individuals that were selected to participate until we reached our intended sample size.

To facilitate an open and comfortable environment for discussion, the focus groups themselves were homogeneous in terms of health care professional type. This approach allowed us to analyze our data within profession type (i.e. surgeon versus surgical trainee) and to compare our findings between the two groups. Any common themes that emerge from variation are particularly relevant to the research question [10].

### Sample size

We included six or seven participants in each focus group for a total sample size of 20 participants plus two interviewees. Our sample provided enough saturation [11] in the data to adequately describe the perceptions about barriers to and facilitators for IPV screening by orthopaedic surgeons and surgical trainees.

### Recruitment

A recent prevalence study conducted at our institution found that the prevalence of IPV (physical, emotional, sexual) among female patients in orthopaedic fracture clinics during a period of 12 months was alarmingly high; 31.6% [4]. As a result, participants for this research were recruited from this fracture clinic, as well as two other fracture clinics in Hamilton, Ontario, all which are affiliated with an academic teaching institution. Individuals who agreed to participate in the focus groups were likely to have an interest in IPV and yield the “information-rich” cases that are important for qualitative research [10]. Potential participants were sent an email invitation which briefly outlined the subject, purpose, agenda and expected outcomes of the focus group. Prior to proceeding with this study, ethics approval was obtained from the local Research Ethics Board (Project Number: 11–491). We asked each participant to sign an informed consent form before proceeding with the focus group or interview. Participants were not financially compensated for participating in this study and they were assured that their participation or lack thereof would not impact their employment or residency status.

### Focus groups

We used a semi-structured focus group guide to structure the discussion about health care professionals’ experiences with and perceptions about screening women for IPV. This approach is useful as it provides participants with some guidance on what to discuss while also enabling exploration of issues that may not have been considered by the researchers [12]. Participants were also asked to complete a brief demographic questionnaire.

The focus groups were conducted in private location at a time convenient for participants and were facilitated by an experienced focus group facilitator (MS). The focus groups were digitally recorded and the recordings were transcribed verbatim.

### Structured interviews

To address institutional and personal barriers such as those identified by the focus group participants, we recognize that it is important to have institutional support and changes to policies at a level above the fracture clinic. We chose to interview two opinion leaders in the field of orthopaedic surgery, who are well-versed and active in health policy in the field of orthopaedic surgery. The purpose of the interviews were to help us to better understand the barriers at the policy level and to identify facilitators for making changes to policy to better assist IPV victims within the fracture clinic setting. The independent, semi-structured telephone interviews were conducted after the completion of the three focus groups which allowed for a more detailed exploration of the themes identified during the focus group discussion. An experienced interviewer (MS) conducted the two interviews which were digitally recorded and transcribed verbatim.

### Data analysis

In qualitative research, data collection and data analysis usually occur simultaneously to allow for new themes in the early data to be incorporated into collection of later data. In this study, we used field notes from the focus groups to identify new themes to explore in future focus groups and we began coding after each focus group. As data collection proceeded, new data and new insights about the data were incorporated into the data analysis, making it reflexive and interactive.

Four investigators (SS, MS, RS, and KM) participated in the data coding and analysis of the focus groups and interviews. When all of the transcripts from the focus groups were coded, the four investigators met to organize the codes into meaningful clusters [13,14] and discussed potential relationships between the categories, a process known as axial coding [15]. The analysis resulted in an organized and comprehensive summary of orthopedic surgeons’ and surgical trainees’ experiences and perceptions related to IPV screening in orthopaedic fracture clinics. The transcripts from the two interviews were coded by the four investigators following the procedures described above.

Transcripts and field notes from the focus groups and interviews were analyzed using conventional qualitative content analysis, as recommended for qualitative descriptive studies [9]. In conventional qualitative data analysis the coding categories are derived directly from the data rather than using preconceived categories [13,14]. The qualitative software program N’Vivo (version 10.0; QSR International, Melbourne, Australia) was used for data management and analysis.

### Rigor

The study’s credibility was ensured by documented evolution of coding and analysis as well as coding decisions [16]. Rigor was also achieved through the use of multiple coders, many with a strong knowledge of the IPV literature, and coding consensus meetings. All categories were firmly grounded in the data by identifying sections of the transcripts from which they originated [17] and quotes were used to illustrate the codes which further demonstrated a good fit between the data and the analytic results.

## Results

### Participants

We invited ten surgeons, ten junior residents, and ten senior residents to participate in the focus groups. Each of our three focus groups included six or seven participants, with a total of 20 surgeons or surgical trainees participating across the three focus groups. The mean age of the focus group participants was 33.9 ± 8.6 years and the majority of the focus group participants were male (75%) (Table 1). The mean length of time in practice for the orthopaedic surgeons was approximately ten years.

**Table 1:** Participant demographics

**Item**	**Number (%)**
	** N = 20**
Age (Mean ± Standard Deviation)	33.9 ± 8.6
Gender	
Male	15 (75%)
Female	5 (25%)
Ethnicity	
Caucasian	11 (55%)
South-East Asian	4 (20%)
Asian	2 (10%)
Native Canadian	1 (5%)
Middle-Eastern	1 (5%)
Other	1 (5%)
Occupation	
Orthopaedic Surgeon	7 (35%)
Surgical Trainee – Senior Orthopaedic Surgical trainee	6 (30%)
Surgical Trainee – Junior Orthopaedic Surgical trainee	7 (35%)
Length of Time in Practice (Orthopaedic Surgeons Only) (Mean ± Standard Deviation)	9.4 ± 9.6

### Themes

Four main themes were identified: contextual thoughts on IPV management, barriers to screening for IPV in fracture clinics, facilitators for screening for IPV in fracture clinics, and policy implementation for fracture clinics. Within the contextual thoughts on IPV, the following two subthemes were identified: perceptions and observations, and comparison to external models in orthopaedics. The focus group discussions yielded additional subthemes that reflected barriers to screening for IPV including fracture clinic barriers, perceived barriers for patients, perceived barriers specific to surgical trainees, and perceived barriers for surgeons. The following subthemes under the overall category of facilitators for IPV screening were identified: system-level characteristics, fracture clinic processes, and personnel resources. The policy implementation theme emerged from the analysis of the two interview transcripts.

### Contextual thoughts about IPV screening in the fracture clinic setting

**Figure 1:**
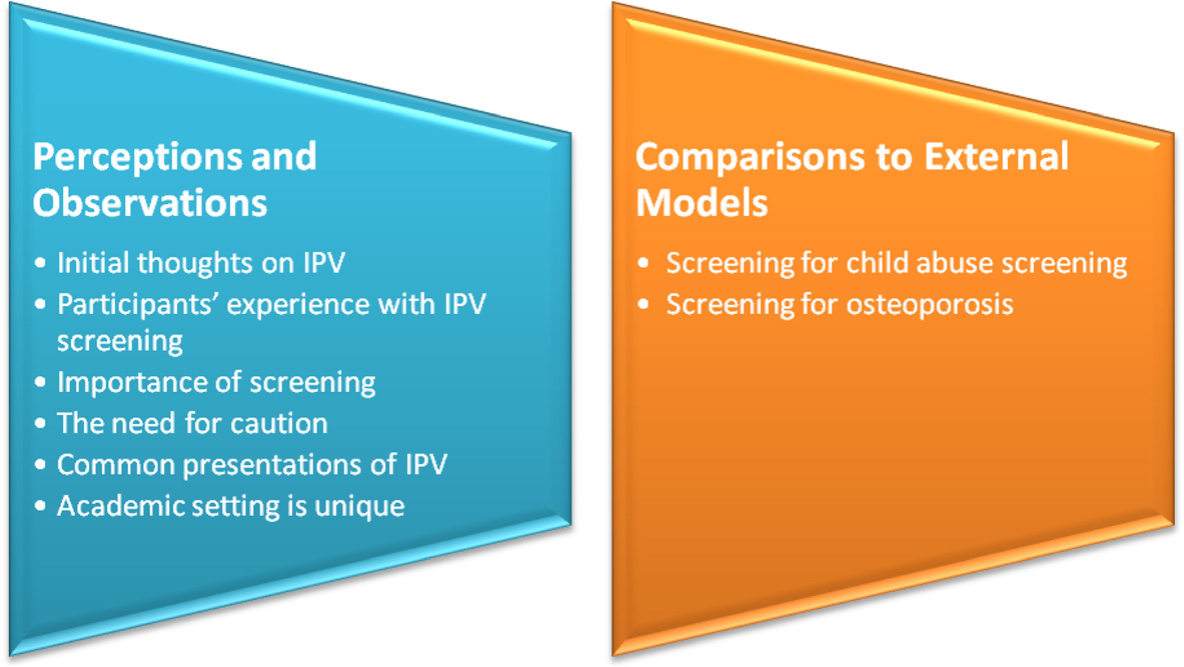
Contextual thoughts on IPV.

The focus groups began by having participants share their initial thoughts on IPV and some of their personal experiences with IPV in the fracture clinic (Figure 1). One focus group began with the following example, “I have a face of intimate partner violence and murder… Two patients, one tried to have his wife killed. She became my patient because they were unsuccessful in killing her”. Most participants recognized and understood the importance of screening for IPV in the fracture clinic setting with one participant noting “I think the screening is hugely important because it probably opens up a door to a certain percentage of women that would then open up and tell you about it” and another describing “I think we are the first access point for those women”. The participants also discussed the need for caution when screening to ensure the safety of the patient.

When discussing perceptions about common presentations of IPV within the fracture clinic setting one participated noted, “… identification is key… that’s the part that I am most worried about is that I am missing people”; while another participant described: “…the biggest problem is picking it [intimate partner violence] up ‘cause I think so much of it is silent”. Another participant summarized current IPV screening practices: “It seems like if we are going on our intuition and our sense that something is wrong, we are doing a bad job with that from the evidence”.

Surgeons and surgical trainees agreed that they are more comfortable with screening for and caring for patients who are victims of child abuse as a result of policies being in place in fracture clinics and having received appropriate training. One participant compared knowing the steps to take after screening for child abuse with not knowing the steps to take after screening for IPV: “With children we sort of know what to do, who to call. There’s like a social work team we call but, you know, if you screen and then they do sort of come forth with “yes it was intimate partner violence” I wouldn’t really know what to do next and so it’s a little intimidating”.

Participants also discussed selective screening for IPV (asking people about IPV based on pre-determined risk factors) versus universal screening for IPV (asking everyone in the fracture clinic about IPV) within the fracture clinic setting. For example, one participant said: “I think it’s more ideal for screening everybody … because if not we are gonna screen people based on our certain assumptions and I don’t think that’s appropriate”. Most participants recognized the limitations and challenges with selective screening and agreed that universal screening for IPV in fracture clinics may be appropriate, citing the success of universal screening for osteoporosis within the fracture clinic setting.

### Fracture clinic barriers to screening for IPV

**Figure 2:**
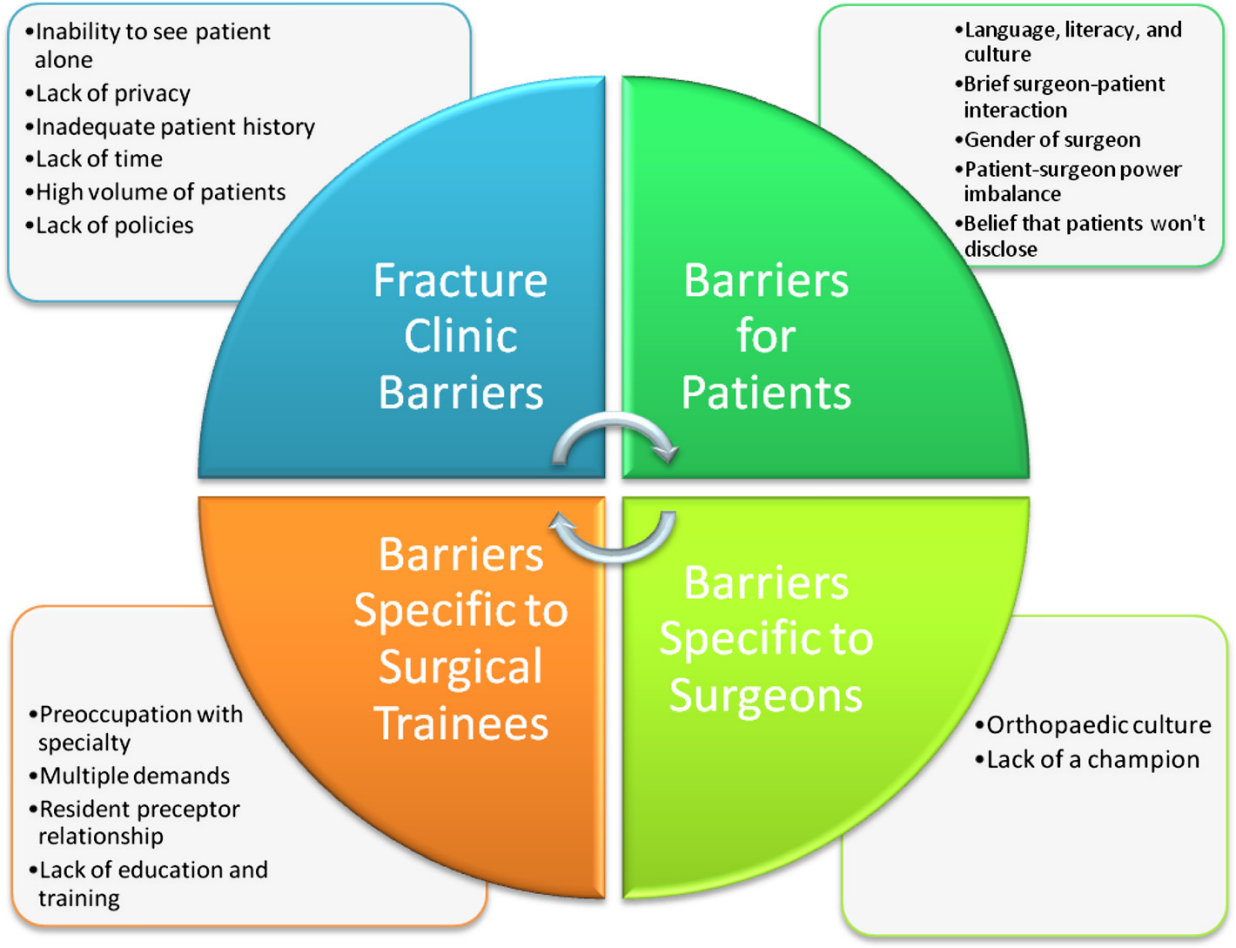
Perceived barriers for screening for IPV in the fracture clinic.

Participants described how the layout and organization of many fracture clinics makes it challenging for the orthopaedic surgeon to have privacy with their patients (Figure 2). Patients are often accompanied by someone to their appointments and it is difficult to separate the patient from this person. In addition, within the academic setting, orthopaedic surgeons rarely see their patients alone as they are usually accompanied by surgical learners when seeing their patients. One participant explained: “There’s six other people, at least six plus learners so probably twelve people listening to every single conversation I have with patients; it’s not the appropriate place”. In addition, many fracture clinics follow an open concept model, with curtains separating exam rooms. One participant made the following analogy: “The fracture clinic is the equivalent of a family doctor seeing patients in their waiting office”.

Focus group participants also identified that adequate patient histories are not readily available or quickly accessible in the fracture clinic and noted that this is a barrier. Since the patient is often initially seen at the emergency department or by a surgical trainee, the surgeon may not be aware of their full history including previous injuries and the nature of these injuries.

Orthopaedic surgeons and surgical trainees described that they spend a very limited time with individual patients and have a high number of patients to see within their fracture clinic, which makes it difficult to find the time to appropriately screen for and address IPV. One participant described: “The biggest thing is time for orthopaedic surgeons. We see 70 to 80 people in a five hour clinic so if you do the math that’s less than a few minutes a person”.

Both the surgeons and surgical trainees identified that there is a lack of policies on screening for and addressing IPV within the fracture clinic setting. The following exchange between the facilitator and several surgeons exemplifies this concern: Facilitator 1- “So am I right in understanding that the clinics you work in have no policies or sort of integrated mechanisms … for screening for intimate partner violence?” Several Surgeons- “None that I’m aware, that’s correct”.

### Perceived barriers for injured patients regarding screening for IPV

The focus group participants were concerned that universal screening may be challenging if the patients are unable to speak, read, and/or write English. They were also concerned about responding appropriately to possible cultural differences and distinguishing cultural practices from signs of IPV.

Another perceived patient barrier identified during the focus groups was the brief interaction that the patient has with the surgeon, and that the short amount of time spent with the patient is focused on the patient’s orthopaedic problem. As one participant explained, “It’s hard to develop a feeling of trust in a short period of time”.

The focus group participants acknowledged that IPV victims may be more comfortable disclosing to females than males, and the field of orthopaedic surgery is predominately male.

Focus group participants recognized that a patient-surgeon power imbalance may be present which could deter patients from disclosing. One participant described this imbalance: “There’s already a power balance right there and then there’s the body language alone and we almost always wear white coats in there. There’s just a lot of overlay that would probably inhibit the patient from disclosing some information”.

There was concern that patients may not want to disclose IPV, as revealed by the following comment: “If someone comes into the fracture clinic they may just want care for their physical injuries”. However, one participant disagreed, stating that “patients tend to trust medical professionals and may want to open up to someone”. Participants also discussed that women may not disclose due to fear of the consequences of disclosing.

### Perceived barriers specific to surgical trainees screening for IPV

In general, the surgical trainee participants felt pre-occupied with their learning and clinical activities and felt that they would not have the focus to screen for IPV. This is demonstrated by the following comment, “You are trying to do a good job at what you are, you know, at becoming a good surgeon and that’s taking a lot of our energy away”.

Surgical trainees have multiple demands on them, as illustrated by the following quote, “We’re busy with our … patient load and our next exam coming up and our … evaluation and our fellowships and our no jobs and our everything else that we think about all the time and … that you are going into the OR the next day and you have to read this tonight….”

The surgical trainees expressed concern about IPV screening influencing their relationship with the preceptor. The focus group participants clearly indicated that their preceptor would have to be supportive and encourage IPV screening.

The focus group participants also expressed concern about the lack of education and training that they received on IPV and consequently they were unsure of what to do with a positive screen. A few participants felt fearful about being held accountable due to lack of ability to identify victims as indicated in the following comment, “you saw Mrs. Smith … she had this injury, it was obviously domestic violence cause she’s dead now and this was her fourth occurrence and you didn’t pick up on it, why not doctor?”.

### Perceived barriers specific to orthopaedic surgeons screening for IPV

The participants felt that the orthopaedic culture, which includes short term interaction with patients and a certain personality-type, is an important barrier. One participant explained, “I think it’s a lot of the perceptions of the surgeon, you know, not that orthopaedics isn’t a caring profession but there are some people that, you know, don’t have the best bedside manner. So I can see people feeling very reluctant to kind of share something personal”. The orthopaedic surgeons also described the belief that screening for IPV falls outside of the role of the orthopaedic surgeon and that they tend to treat the injury in isolation, as opposed to treating the social and other medical issues.

Focus group participants indicated that there is lack of a champion for promoting IPV screening and effective social support for IPV victims, as described in the following comment, “There’s no champion right now who actually works at the fracture clinic”.

### System-level characteristics that are facilitators for screening for IPV in the fracture clinic setting

**Figure 3:**
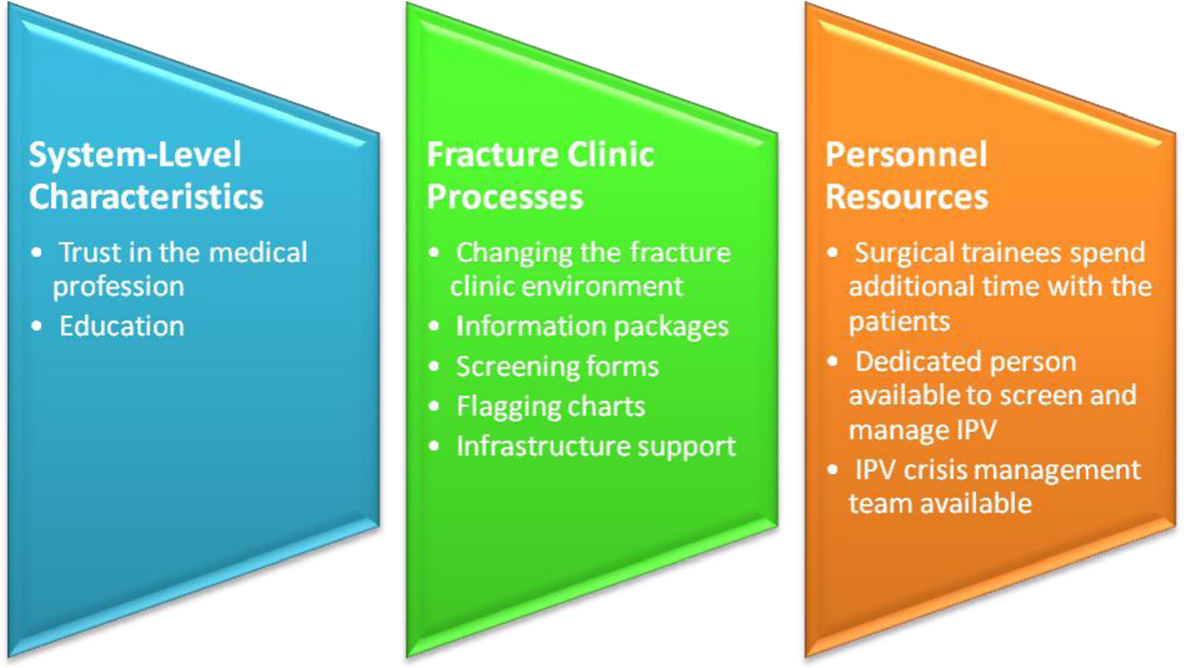
Facilitators for screening for IPV.

The participants indicated that trust of the medical profession was an important facilitator, as patients often open up to medical professionals (Figure 3). One of the participants commented, “I’m sometimes surprised at how open and forthcoming patients are in the short time you get to know them the things that they’ll tell you. I mean I think there is a sort of inherent trust in the medical profession”.

The focus group identified education for surgeons and surgical trainees as an important facilitator, as described in the following comment, “I think the next step would be towards education and make it not just completely academic but with these things I think you have to have case scenarios…”

### Changes to fracture clinic processes that would facilitate screening for IPV in the fracture clinic

The focus group participants suggested numerous changes to the fracture clinic environment to facilitate IPV screening and care for IPV victims. Examples described included having a private area where surgeons could talk to patients about confidential issues and having posters and pamphlets on IPV available within the fracture clinic to potentially help patients feel more comfortable with the topic.

Participants noted that having a prepared information package for surgeons would greatly assist in screening for IPV. One participant suggested “having something like a preprinted order pack, an abuse care package so that I know, not just academically what resources are available … it has resources for the patient that has a set of orders”.

Another idea that was raised during the focus groups with having a screening form which the patient would complete upon presentation to the fracture clinic which could help to identify high risk patients for the orthopaedic team to follow-up with.

The focus group participants suggested that they could flag the charts of potential IPV victims. The following participant describes this suggestion: “They can stick like a red dot on the chart or something and then we go okay maybe that red dot means that we should ask them …”

The focus group discussions included suggestions for changing the infrastructure within the fracture clinic to facilitate screening for IPV. These suggestions include ensuring that there is adequate support from all fracture clinic staff and the hospital.

### Personnel resources for facilitators for screening for IPV in the fracture clinic setting

Focus group participants suggested that surgical trainees have the opportunity to spend more time with patients so that they can effectively screen for IPV, as suggested in the following comment: “I think sometimes …patients might actually disclose [IPV] to surgical trainees more than surgeons because surgical trainees tend to spend more time with the patient in the fracture clinic in terms of taking the history and doing the physical exam”.

There was a great deal of discussion on the need for a dedicated person within the fracture clinic setting to screen for and manage IPV. The following quote from a surgical trainee demonstrates this: “When we were doing the screening for the IPV Prevalence study we had, you know, students coming in and asking every patient “can I talk to you in private?” That did it quite effectively”.

Facilitators for caring for IPV victims included having a simple plan in place for the fracture clinic including the availability of a crisis team. A participant noted, “You should give them a simple plan that this is a person to contact when these are the problems in the fracture clinic or this is the person coming to the clinic every day three four hours and in case you find some clue you don’t need to waste much time because you are busy”.

### Policy implementation

**Figure 4:**
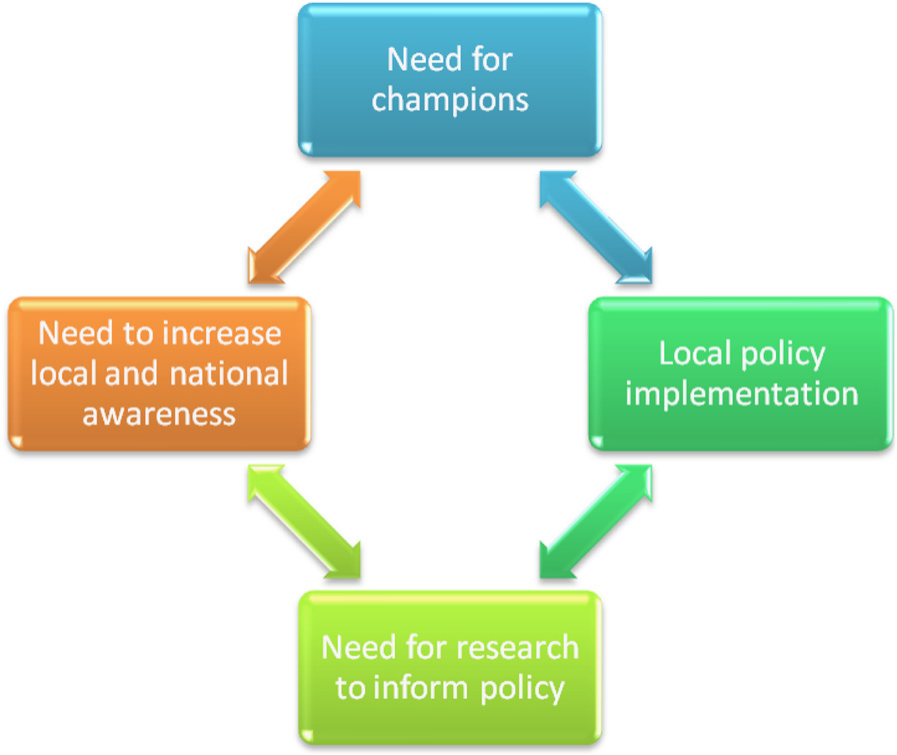
Implementation of policies for IPV screening.

Both opinion leaders were asked about the need for local and national champions to promote the need for IPV screening and care for victims within the fracture clinic setting (Figure 4). Both believed that a champion would be invaluable, as evident from the following quote: “I think, you always need a champion not only to develop the policy but also to implement the policy”.

The interviewees identified a need to increase awareness about IPV among all health care professionals working within the fracture clinic setting. One opinion leader explained, “…the data that shows in the last year 30% of women that present to a fracture clinic have been subject to some type of IPV and I’m not so sure that that people even know those numbers so I think, you know, getting the information out there that it impacts a huge segment of the population, that the impact is substantial …”

The interviewees agreed that local policy on IPV would be beneficial in assisting patients who experience abuse. As one opinion leader stated, "…it’s easier taking a grassroots approach, you know, go to a smaller entity like a hospital or clinic…if you have enough you know clinics that a or hospitals that are sort of buying into this program which is being run more at a grassroots level then maybe at the sort of provincial or systemic level someone might so oh this is working really well or seems to be really important, what we need to do is formalize this".

The need for research to inform policy was also discussed and both opinion leaders believed that the implementation of an IPV screening program should be evidence-based and they supported future research to better inform the decision makers.

## Discussion

The present study aimed to identify barriers to and facilitators for screening and caring for IPV victims in an orthopaedic fracture clinic setting. The current study confirmed that many of the barriers to screening for IPV in other medical specialties are also present within the fracture clinic setting. In addition, this study identified several additional barriers and facilitators that are specific to orthopaedic surgeons, orthopaedic surgery trainees, and the fracture clinic environment. Briefly, Sormanti & Smith and Colarossi et al. conducted focus groups with emergency department surgical trainees and health care staff at an urban family planning centre to examine the perceived barriers to screening for IPV in these specific health care settings. Similar to our findings, they reported barriers to IPV screening that included time constraints, unpreparedness to screen for IPV and discuss the issue comfortably and thoroughly, lack of clarity about implementation of screening and inadequate referral resources [5,7]. Waalen et al. conducted a review of published studies reporting on barriers to IPV screening among various health care professionals [18]. They found the following provider-related barriers: a lack of provider education regarding IPV, lack of time, and lack of effective interventions. The authors also identified the following patient-related factors: patient nondisclosure and fear of offending the patient. This review also reported that barriers to screening for IPV are documented to be similar among health care providers across diverse specialties and medical settings. Waalen et al. did not include any studies in the field of orthopaedic surgery [18]. A recent systematic review investigating barriers to IPV screening reported by a variety of health care professionals found many barriers that were also identified in the current study such as a lack of time, language and cultural barriers, lack of training, and lack of institutional protocols [8]. The current study identified several barriers that were specific to orthopaedics that the systematic review did not report in other specialties. These include lack of a “champion”, the male-dominated nature of orthopaedics, orthopaedic culture, and the perception of a patient-surgeon power imbalance. Interestingly, none of our focus group participants noted that personal discomfort with IPV, fear of offending patients, or the perception that abuse is rare are barriers to IPV screening, as were commonly noted in other specialties [8].

The published literature demonstrates an exploration of the factors that influence IPV screening in multiple areas of health care, although none of the previous literature focuses on orthopaedic fracture clinics. Our previous research in the field of IPV and orthopaedics has found that injuries requiring the consultation of an orthopaedic surgeon account for 28 percent of clinical manifestations of IPV, but IPV is underemphasized in this medical specialty [3]. A recent survey of orthopaedic surgeons reported that nine percent of the respondents believed that inquiring about IPV was an invasion of the patient’s privacy, and eleven percent believed that ruling out IPV as the cause of injury was not part of their duty [3]. The current study further explored these biases and identified barriers to screening for and addressing IPV in the orthopaedic fracture clinic. Many of the barriers identified in the current study can be addressed with education and the appropriate resources and infrastructure. For example, surgeons and trainees can learn how to approach the topic with sensitivity and confidence in their knowledge of the appropriate plan to take in the event that a patient discloses. Additionally, fracture clinics can be designed or renovated to reflect a more private environment that is conducive to discussing sensitive topics such as a patient’s experience with IPV.

Our focus groups found that the participants were, in general, supportive of implementing practices to improve IPV screening and care within the orthopaedic fracture clinic setting. They identified numerous facilitators, which could be implemented into orthopaedic fracture clinics to help effectively identify and help IPV victims. After analyzing our codes from the three focus group transcripts, it was apparent that there were many shared opinions, attitudes, and experiences among participants. For example, a common theme identified in each focus group discussion was a lack of time and privacy. However, some of the barriers identified by the surgical trainees were unique because they did not apply to the practicing orthopaedic surgeons (e.g. surgical trainee preceptor relationship). There were no other differences in the responses between groups. Our interviews with opinion leaders confirmed the findings from the focus groups and provided insight into and suggestions for implementing policies for IPV screening and providing health care professionals with the structure and support to effectively care for IPV victims.

Our methodology aimed to reduce bias and yield a purposeful sample. The qualitative descriptive method chosen for this research minimizes researcher biases as this method involves minimal interpretation of data and focuses on presenting and organizing the data in the language used by participants [19]. Recruiting volunteers to participate likely led to a bias of having individuals who are interested in the topic of IPV screening participate in our research. Since the goal in purposeful sampling is to include participants who can provide “information-rich” data [10], this bias is favorable as participants who have an interest in IPV screening are likely able to provide detailed information, experiences and perceptions about perceived barriers to and facilitators for IPV screening. In addition, the questions within the focus group guide were worded to ensure that they were neutral and did not lead the participants. The focus group facilitator used neutral probes and reflective statements to clarify what had already been discussed and to encourage more discussion among participants. The use of homogenous groups of professionals ensured that participants within each focus group had similar education levels and clinical expertise which helped to minimize bias related to conforming and suppressing minority views that might be different from views of dominant participants within the group.

This study is limited by a relatively small sample size and by having all focus group participants affiliated with one academic institution. We are also limited by having only interviewed two opinion leaders and therefore, the results of these interviews should be interpreted cautiously. The two opinion leaders were from different academic institutions which may improve the generalizability of our findings, and it should be noted that there are few opinion leaders in the field of orthopaedics in Canada who are well-versed on the topic of IPV and health policy. It is important to note that there may have been bias in deriving our focus group sample exclusively from one academic institution because this community of health care professionals and trainees has had previous exposure to IPV through education initiatives to increase awareness and sensitivity to the issue. Additionally, researchers at this institution have published research focusing on IPV within the field of orthopaedic surgery. This unique culture may have influenced the attitudes and opinions of the orthopaedic surgeons and surgical trainees and this may limit the external validity of our results. This study warrants replication in other jurisdictions and in a community setting to further determine the generalizability of the findings. Future research could also investigate patients’ opinions of screening and caring for IPV victims within the orthopaedic fracture clinic setting.

## Conclusions

There are a number of perceived barriers to screening women in the fracture clinic for IPV, many of which can be addressed through increased education and training, and additional resources in the fracture clinic. Orthopaedic health care professionals are supportive of implementing an IPV screening program in the orthopaedic fracture clinic.

## Competing interests

The authors declare that they have no competing interests.

## Authors’ contributions

All authors participated in the study conception and design. MS, KM, and RS participated in data collection. All authors participated in data analysis and manuscript preparation. BAP and MB secured study funding. All authors read and approved the final manuscript.

## Pre-publication history

The pre-publication history for this paper can be accessed here:

http://www.biomedcentral.com/1471-2474/14/122/prepub
